# Enhancement of Phosphorylation and Transport Activity of the Neuronal Glutamate Transporter Excitatory Amino Acid Transporter 3 by C3bot and a 26mer C3bot Peptide

**DOI:** 10.3389/fncel.2022.860823

**Published:** 2022-06-15

**Authors:** Johannes Piepgras, Astrid Rohrbeck, Ingo Just, Stefan Bittner, Gudrun Ahnert-Hilger, Markus Höltje

**Affiliations:** ^1^Department of Neurology, Focus Program Translational Neuroscience and Immunotherapy, Rhine-Main Neuroscience Network, University Medical Center of the Johannes Gutenberg University Mainz, Mainz, Germany; ^2^Institute of Toxicology, Hannover Medical School, Hanover, Germany; ^3^Department of Neurobiology, Max Planck Institute for Biophysical Chemistry, University of Göttingen, Göttingen, Germany; ^4^Institut für Integrative Neuroanatomie, Charité – Universitätsmedizin Berlin, Corporate Member of Freie Universität Berlin and Humboldt-Universität zu Berlin, Berlin, Germany

**Keywords:** C3 transferase, glutamate transporter, EAAT3, phosphorylation, uptake

## Abstract

In primary murine hippocampal neurons we investigated the regulation of EAAT3-mediated glutamate transport by the *Clostridium botulinum* C3 transferase C3bot and a 26mer peptide derived from full length protein. Incubation with either enzyme-competent C3bot or enzyme-deficient C3bot^156–181^ peptide resulted in the upregulation of glutamate uptake by up to 22% compared to untreated cells. A similar enhancement of glutamate transport was also achieved by the classical phorbol-ester-mediated activation of protein kinase C subtypes. Yet comparable, effects elicited by C3 preparations seemed not to rely on PKCα, γ, ε, or ζ activation. Blocking of tyrosine phosphorylation by tyrosine kinase inhibitors prevented the observed effect mediated by C3bot and C3bot 26mer. By using biochemical and molecular biological assays we could rule out that the observed C3bot and C3bot 26mer-mediated effects solely resulted from enhanced transporter expression or translocation to the neuronal surface but was rather mediated by transporter phosphorylation at tyrosine residues that was found to be significantly enhanced following incubation with either full length protein or the 26mer C3 peptide.

## Introduction

The prototypical *Clostridium botulinum* C3 transferase (C3bot) is an exoenzyme that represents a family of bacterial ADP-ribosyltransferases that, when taken up into target cells, effectively block Rho-signaling in an enzymatic manner (Aktories and Frevert, 1987; Aktories and Just, 2005). Small GTPases of the Rho family, especially RhoA, serve key functions as molecular switches in the regulation of the cytoskeleton in general and also in neuronal cells, and C3 protein has been extensively used as tool to investigate growth- and regeneration-associated morphological processes in different types of neurons. Activated RhoA signaling executed by downstream effector kinases like ROCK leads to phosphorylation of myosin light chain thereby inducing growth cone collapse and axon retraction as observed after ischemic or traumatic brain injuries (Mulherkar et al., 2017; Sladojevic et al., 2017; Takase and Regenhardt, 2021). In this context, by using full length C3bot or C3-derived peptides (that lack the specific enzymatic activity of full length protein), our group and others have demonstrated the beneficial outcome of a treatment with C3bot for neuronal re-growth following damage of the CNS. This includes experimental lesion models both *in vitro* and *in vivo* (Dubreuil et al., 2003; Höltje et al., 2009; Boato et al., 2010; Loske et al., 2012) as well as clinical trials in patients with spinal cord injuries (McKerracher and Anderson, 2013; Watzlawick et al., 2014; Fehlings et al., 2018). For a long time unknown, recent work from our group has identified the intermediate filament protein vimentin as well as β1-integrin as cellular receptors for C3bot (Rohrbeck et al., 2014, 2017; Adolf et al., 2019). As mentioned, growth-promoting effects are also caused by enzyme-deficient C3bot-derived peptides like the 29mer C3bot^154–182^ or 26mer C3bot^156–181^, both including the catalytic amino acid glutamate at position 174 of full length C3bot, but in this case, the exact cellular enzyme-independent mechanisms remain to be unraveled.

However, besides exhibiting morphological effects C3bot also affects cellular transmitter transport mechanisms. Incubation of astrocytes with C3bot results in a NFκB-dependent upregulation of glial high-affinity glutamate transporter EAAT2 (GLT-1) accompanied by increased glutamate uptake. At the same time astrocytes treated with C3bot released higher amounts of glutamate by vesicular release (Höltje et al., 2008). Prompted by these findings we investigated putative effects of C3bot and enzyme-deficient C3bot^156–181^ (C3bot 26mer) on glutamate uptake of hippocampal neurons.

As long known, glutamate not only represents the major excitatory neurotransmitter of the CNS but can also cause neuronal excitotoxicity at higher extracellular levels (Choi and Rothman, 1990; Lipton and Rosenberg, 1994). Therefore, maintenance of physiological extracellular glutamate levels is pivotal for fidelity of synaptic transmission and even to prevent neuronal cell death. Clearance from the synaptic cleft is mediated by high affinity glutamate transporters. Though not exclusively expressed at the cell surface, the neuronal excitatory amino acid transporter 3 (EAAT3) belongs to a family of five potassium-dependent glutamate transporters located at the plasma membrane (Arriza et al., 1997). Generally, glutamate transport is coupled with sodium-dependent H^+^ inward-transport and counter-transport of K^+^ (Owe et al., 2006). Besides their role in transporting amino acids, EAATs also function as chloride channels (Wadiche et al., 1995).

While the majority of glutamate clearance in the CNS is mediated by the mainly astroglial EAAT2, postsynaptic EAAT3 activity rather affects local glutamate concentrations and neighboring receptors (Lehre and Danbolt, 1998; Holmseth et al., 2012; Bjørn-Yoshimoto and Underhill, 2016). Moreover, EAAT3 provides the source of glutamate as precursor for GABA synthesis in inhibitory neurons (Mathews and Diamond, 2003) and therefore is involved in controlling inhibitory signaling in the brain. Neuronal EAAT3 plays another crucial role in preventing vulnerability to oxidative stress and to maintain redox homeostasis. Cysteine is an alternative transport substrate for EAAT3 and represents the rate-limiting factor for glutathione synthesis needed to reduce reactive oxygen species such as H_2_O_2_ (Griffith, 1999). In line with this, perturbation of EAAT3 surface transport activity is associated with various neuronal pathologies, including Parkinson’s disease, epilepsy, or Huntington’s disease (for review, see Malik and Willnow, 2019).

C3 proteins have proven to foster axon re-growth and to provide neuroprotection under pathological conditions that typically involve a disturbed glutamate balance in the CNS. Therefore, the current study was undertaken with the following aims: By using primary hippocampal neuronal cultures we investigated the effects of C3bot on neuronal glutamate uptake. Furthermore, we determine whether the effects strictly rely on enzyme-activity of C3 (as already shown for morphological and functional effects on astrocytes) or can also be elicited by enzyme-deficient C3bot 26mer.

Last but not least we analyzed the regulatory mechanisms underlying functional regulations of EAAT3 mediated by C3bot and C3bot 26mer.

## Materials and Methods

### C3 Proteins

Development of C3bot-derived peptide and full length protein:

C3bot^156–181^ (C3bot 26mer), was synthesized at Pharis Biotec GmbH (Hannover, Germany). C3bot was expressed as recombinant GST-fusion protein in *E. coli* TG1 harboring the respective DNA fragment in the plasmid pGEX-2T (Höltje et al., 2009).

### Antibodies

#### Immunofluorescence

A polyclonal antibody against the excitatory amino acid transporter 3 (EAAT3) was purchased from Santa Cruz Biotechnology (#sc-25658, Santa Cruz, TX, United States). Polyclonal antisera obtained from Chemicon (Temecula, CA, United States) were used for detection of glutamate-aspartate transporter EAAT1 (GLAST, #AB1782) and glial glutamate transporter EAAT2 (GLT-1, #AB1783). Protein kinase C-alpha (PKCα) was detected by a monoclonal antibody obtained from BD Biosciences (#610107, Heidelberg, Germany). A polyclonal antibody against actin was purchased from SIGMA (#A5060 St. Louis, MO, United States). Morphology of hippocampal neurons was visualized by a polyclonal antiserum against microtubule associated protein 2 (MAP2, #AB5622) and neurofilament protein of 200 kDa (#AB5256) from Chemicon International (Hofheim, Germany). An affinity purified polyclonal rabbit IgG against full length C3bot developed by our group was applied (Rohrbeck et al., 2014). To detect the phosphorylation levels of EAAT3 a polyclonal antibody directed against phosphotyrosine was purchased from Santa Cruz Biotechnology (#sc-7020). Antibodies against protein kinase C isoforms γ (monoclonal #ab71558), ε (polyclonal #ab63638), and ζ (polyclonal #ab108970) were obtained from Abcam (Cambridge, United Kingdom).

#### Immunoblotting

Excitatory amino acid transporter 3 as well as actin antibodies were the same as used for immunofluorescence. Polyclonal anti EAAT1/GLAST (agc-021) was purchased from Alomone Labs (Jerusalem, Israel) and polyclonal anti EAAT2/GLT-1 was purchased from Abcam (Waltham, MA, United States). Monoclonal anti RhoA was purchased from Santa Cruz Biotechnology (#26C4). For detection of phosphorylated serine a polyclonal antibody from Abcam was used (#ab9332, Cambridge, United Kingdom). Detection of phosphotyrosine was achieved by using the same antibody as used for immunofluorescence.

### Cell Culture

#### Hippocampal Cell Culture

Neuronal cultures from hippocampi of NMRI or SWISS mice were prepared from fetal animals at embryonic day 16 (E16). Dissected tissue pieces were rinsed with PBS, then with dissociation medium (MEM supplemented with 10% fetal calf serum, 100 IE insuline/l, 0.5 mM glutamine, 100 U/ml penicillin/streptomycin, 44 mM glucose and 10 mM HEPES buffer) and dissociated mechanically. Spun-down cells were resuspended in starter medium (serum-free neurobasal medium supplemented with B27, 0.5 mM glutamine, and 25 μM glutamate) and plated at a density of 8 × 10^4^ cells/well on glass cover slips pre-coated with poly-L-lysine/collagen. All ingredients were obtained from Gibco/BRL. After 12–14 days in culture C3 preparations were added to the culture medium for another 72 h.

#### Astrocyte Culture

Primary astrocyte cultures were prepared from NMRI mice brains between postnatal days 2 and 3. Meninges were removed from whole brains and mechanically dissociated in Hank’s buffered salt solution (HBSS) by fire polished Pasteur pipettes and centrifuged at 300 × *g* for 3 min. Astrocytes were redissociated in HBSS and the procedure was repeated two times using smaller pipette tip diameters. Cells were first seeded onto 6-well plates (3.5 cm diameter/well) pretreated with poly-L-lysine (100 μg/ml in PBS). Astrocytes were incubated at 5% CO_2_ in Dulbecco’s Modified Eagle Medium (DMEM), supplemented with 10% fetal calf serum, 100 U/ml penicillin/streptomycin and 2 mM L-glutamine. Microglial cells were repeatedly detached from the astrocyte monolayer by shaking off. After 7 days in culture with a change of medium for two times cells were harvested and recultured in 24-well plates at a density of 4 × 10^4^ cells/well with glass coverslips pretreated with poly-L-lysine if used for immunofluorescence methods.

### Brain Homogenates

Brains of adult mice were homogenized using a glass/Teflon homogenizer (Wheaton, Potter-Elvehjem, clearance 0.1–0.15 μm) in ice-cold buffer containing 320 mM sucrose, 4 mM HEPES/KOH, pH 7.4, 1 mM PMSF, and 1 μl/ml protease inhibitor cocktail (Pi, Sigma-Aldrich) and centrifuged for 10 min at 4°C and 1300 × *g* (Beckman rotor TLA-100.4). The resulting supernatant was used for detection of EAAT3 by Western blotting.

### Immunohistochemistry and -Cytochemistry

Perfusion-fixed brains from adult NMRI-mice were dissected, cryoprotected and frozen at −80°C prior to cutting into 20 μm coronar sections. The sections were washed with PBS and incubated in blocking solution (10% NGS in PBS; 0.3% Triton-X-100) for 30 min at room temperature. Primary antibodies were applied overnight at 4°C. After incubating the sections, they were washed with PBS and incubated with secondary antibodies for 1 h at room temperature in the dark. Immunoreactivity was visualized using Alexa Fluor 488 or Alexa Fluor 594 goat anti-mouse and goat anti-rabbit secondary antibodies (Molecular Probes, Eugene, OR, United States). Again, sections were washed with PBS, stained with DAPI (4′,6-diamidino-2-phenylindole) for 10 min and mounted with Immu-Mount (Thermo Fisher Scientific, Waltham, MA, United States).

Cultured cells were fixed with 4% formaline for 15 min and subsequently permeabilized for 30 min at room temperature (RT) using 0.3% Triton X-100 dissolved in PBS. Staining with primary antibodies was performed overnight at 4°C. After washing in PBS secondary antibodies were applied for 1 h at room temperature (RT). Immunoreactivity was visualized using secondary antibodies as given above. Fluorescence was either visualized using an upright Leica DMLB epifluorescence equipment or by using confocal laser scanning microscopy (see below).

### Confocal Laser Scanning Microscopy

Images were acquired using a Leica TCS SL confocal laser scanning microscope using a x40 oil immersion objective. Fluorescent dyes were excited at a wavelength of 488 nm (green fluorescence) and 543 nm (red fluorescence), respectively. Fluorescence from green and red channels was sequentially collected using two filters at 498–535 nm and 587–666 nm, respectively. Images were captured at a resolution of 1024 × 1024 pixels.

### Glutamate Uptake Assay

#### Radiolabeled Glutamate

[^3^H]radiolabeled L-glutamate (specific activity: 20 Ci/mmol) was obtained from Hartmann Analytic GmbH (Braunschweig, Germany). Usually, for glutamate uptake hippocampal neurons grown in 24-well plates were incubated with 50 μM of glutamate (applied as a combination of both tritiated and unlabeled glutamic acid) for 1 h with or without additives as indicated at 37°C in serum-free culture medium. For kinetic analysis of transport activity the uptake time was shortened to 10 min. GraphPad Prism software (San Diego, CA, United States) was used for non-linear regression and transformation of data. After removal of the medium and repeated washing (3x) with ice-cold Krebs-Hepes buffer containing 140 mM NaCl, 4.7 mM KCl, 2.5 mM CaCl_2_, 15 mM Hepes, and 1.2 mM MgSO_4_ adjusted to pH 7.4 cells were lysed with 0.4% Triton X-100 for 10 min at 42°C. Neuron lysates were subjected to liquid scintillation counting for determination of radioactivity. Values were adjusted to protein content.

#### Fluorometric Glutamate Measurements

For fluorometric glutamate detection the glutamate assay kit #ab138883 from Abcam was used according to the manufacturer. In brief, cells were incubated for 1 h with 50 μM of glutamate in serum-free culture medium. Following two washing steps, cells were lysed in 0.4% Trion-X100 as above. Neuron lysates were subjected to fluorometric reading using Ex/Em = 540/590 nm for determination of intracellular glutamate concentrations calculated from standards. Values were adjusted to protein content. The tyrosine kinase inhibitors Dasatinib (ab142050) and Genistein (ab120112) were purchased from Abcam (Waltham, MA, United States).

### Biotinylation Assay

All work steps except for centrifugation in the cell culture centrifuge were carried out in the cold at 4°C or on ice according to manufacturers instructions (Thermo Fisher Scientific, Waltham, MA, United States). Hippocampal neurons cultured in 6-well plates were washed three times with PBS and then incubated with Sulfo-NHS-Biotin solution (1 mg/ml) for 30 min. Thereafter, the cells were washed twice with quenching buffer and then incubated with quenching buffer for 30 min with agitation. Following washing, cells were spun down and pellets obtained were lysed. Avidin agarose beads were added to the lysate and incubated overnight with agitation. The next day, part of the supernatant was saved and spun down avidin beads were washed three times with PBS. The supernatant and avidin beads were taken up in Laemmli buffer and processed for Western blot analysis.

### Immunoisolation and Phosphorylation of Excitatory Amino Acid Transporter 3

Dynabeads^®^ Protein G (Invitrogen) were incubated in antibody binding and washing buffer (PBS with 0.1% Tween-20) containing the antibody of interest. A polyclonal anti-EAAT3 antibody (Santa Cruz Biotechnology) and normal rabbit IgG for control were used in a concentration of 80 ng/ml antibody for binding to the beads (1–2 μg antibody per 200–500 μg of protein in cell lysate, see below). Binding was performed for 1 h at room temperature. Afterward, the antibody-beads complex was washed once in antibody binding and washing buffer and kept on ice. Cells were washed twice in ice cold PBS, scraped off the culture dish and collected in a vessel on ice. After centrifugation at 3000 rpm for 4 min the cell pellet was resuspended in ice cold PBS. After a final centrifugation at 20000 × *g* for 3 min the cell pellet was kept on ice for lysis and protein determination. Cells were lysed in an extraction buffer (KCl 140 mM, Hepes 2 mM and EDTA 20 mM at pH 7.4 together with 1% Triton X-100, 1 mM PMSF, 1 μl/ml protease and phosphatase inhibitors) for 1 h at 4°C. After, the insoluble fraction was separated by centrifugation at 2700 × *g* for 5 min. The supernatant was incubated with the Dynabeads^®^-antibody-complexes over night at 4°C. A part of the supernatant was boiled in Laemmli buffer. The Dynabeads-antibody-antigen-complex was washed three times in extraction buffer without Triton and resuspended in washing buffer before boiling in Laemmli buffer. Phosphorylation status was probed by using anti-phosphotyrosine and anti-phosphoserine antibody after protein separation by gel electrophoresis.

### Imaging Analysis

To quantify the phosphorylation status of EAAT3 by immunofluorescence methods double stainings against EAAT3 and phosphotyrosine (pTyr) were applied to hippocampus cultures grown for 12–14 days *in vitro* and incubated for further 72 h with C3 preparations. Randomly chosen images of individual neurons were acquired at 100x magnification and mean brightness values within a circular region of interest (50 μm in diameter) centered on the soma of the respective neurons were calculated for red and green fluorescence by the histogram tool of Adobe Photoshop CS6. Both channels were superimposed and remaining brightness and saturation values for red and green fluorescence were set to zero (black). Then again, mean brightness values for the remaining yellow fluorescence representing the co-localized signal between EAAT3 and pTyr were calculated.

### Experimental Design and Statistics

Typically, experiments were run at least three times if not stated otherwise. Uptake assays were determined in triplicates or quadruplicates for a single condition. In general, values are expressed as means ± SEM from one representative experiment or pooled experiments. Statistical significance or absence from that was calculated in Figures 3–6 using Student’s *t*-test and *p* values below 0.05 were considered significant.

## Results

**FIGURE 1:**
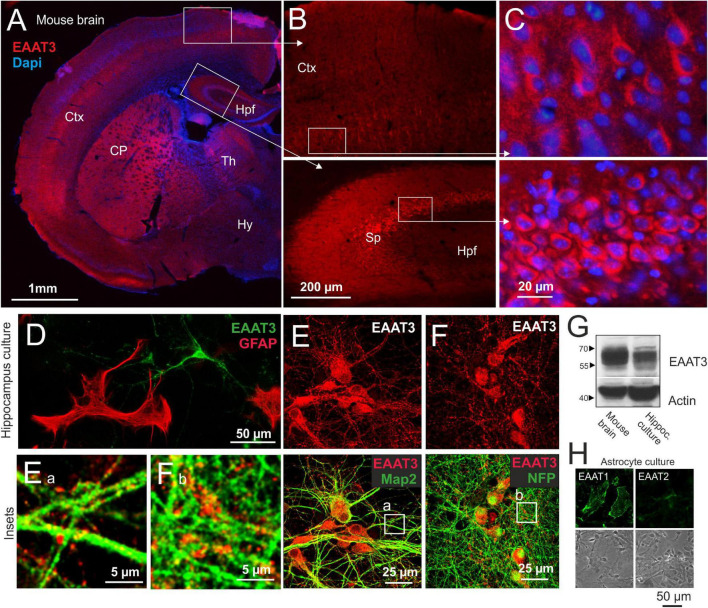
Expression of the excitatory amino acid transporter 3 (EAAT3) in mouse brain and primary hippocampal neurons. **(A–C)** Expression pattern of EAAT3 in the adult mouse brain. Coronar cryosections of adult mouse brains were incubated with a polyclonal antibody directed against EAAT3. Protein expression was detected in neuronal perikarya and the neuropil of various brain areas, including caudate putamen, cortex and the hippocampus formation. Insets show enlargements of depicted areas. CP, Caudate putamen; Ctx, Cortex; Hpf, Hippocampus formation; Hy, Hypothalamus; Sp, Stratum pyramidale; Th, Thalamus. **(D)** Mouse hippocampal neurons were prepared at embryonic day 16, cells were grown on glass cover slips for 14 days, fixed and stained for EAAT3 and GFAP as marker for astrocytes present to some extent in culture. EAAT3 expression was restricted to neurons. **(E,F)** Mouse hippocampal neurons were prepared and cultured as described, fixed and stained for EAAT3, Map2 as dendritic **(E)** and NFP as axonal marker **(F)** by immunofluorescence methods and confocal imaging. EAAT3 staining was observed in a punctuate fashion throughout the cell body as well as dendritic and axonal processes [lower panels, see also insets **(E_a_,F_b_)**]. **(G)** Homogenates of whole mouse brains and cultured hippocampal neurons were subjected to Western blot procedure and probed for the expression of neuronal EAAT3. Actin was used as loading control. The antiserum detected a major band between the 55 and 70 kDa markers in both samples. Overall, the signals corresponded well with the expected molecular weight. **(H)** Murine astrocytes were cultured close to confluency, fixed and stained for the main glial glutamate transporter EAAT1 (GLAST) and EAAT2 (GLT-1) by immunofluorescence and confocal imaging. Astrocytes predominantly expressed EAAT1 at the plasma membrane. EAAT2 expression was observed at comparably low levels.

To investigate C3 protein-mediated effects on neuronal plasma membrane glutamate transporter activity we used primary neuronal cultures from the hippocampus of embryonic mice representing a mixed culture of different neuronal subpopulations. Albeit not exclusively expressed at the plasma membrane, EAAT3 is the main neuronal surface glutamate transporter in the brain (Bjørn-Yoshimoto and Underhill, 2016). It is abundantly expressed *in vivo* in neuronal somata and neuropilar regions of various brain areas including cortex, basal ganglia and the hippocampus formation as shown for the adult mouse brain (Figures 1A–C). The observed distribution corresponded very well with early pioneering studies on its location (Rothstein et al., 1994). To test for suitability of our model system we aimed to evaluate the expression of EAAT3 in the neuronal cell culture used. Following cultivation for 14 days cells were stained for EAAT3 and glial fibrillary acidic protein (GFAP) to confirm neuronal expression of EAAT3. Indeed, transporter expression was clearly attributable to cells morphologically identifiable as neurons (Figure 1D). Further immunofluorescence analysis revealed a strong punctuate distribution of the transporter at the somatodendritic compartment (Figure 1E, microtubule-associated protein 2 was used as marker protein) and axonal processes (Figure 1F, neurofilament protein of 200 kDa was used as marker protein). Transporter signals localized both at surface and cytoplasm which corresponds well with the different functions of EAAT3 served depending on its cellular localization (Bjørn-Yoshimoto and Underhill, 2016). Western blot studies detected a strong major and few weaker bands between 55 and 70 kDa in mouse brain homogenate and hippocampus culture yielding biochemical evidence for the presence of EAAT3 in both preparations (Figure 1G). Presence of astrocytes in neuronal cultures is unavoidable and therefore a glial contribution to observed glutamate uptake must taken into account and should be blocked pharmacologically to analyze pure neuronal transport activity. The basic possibility of glial glutamate transporter expression *in vitro* was confirmed by immunofluorescence microscopic analysis detecting mainly EAAT1 and low EAAT2 expression in purified astrocyte culture (Figure 1H).

**FIGURE 2:**
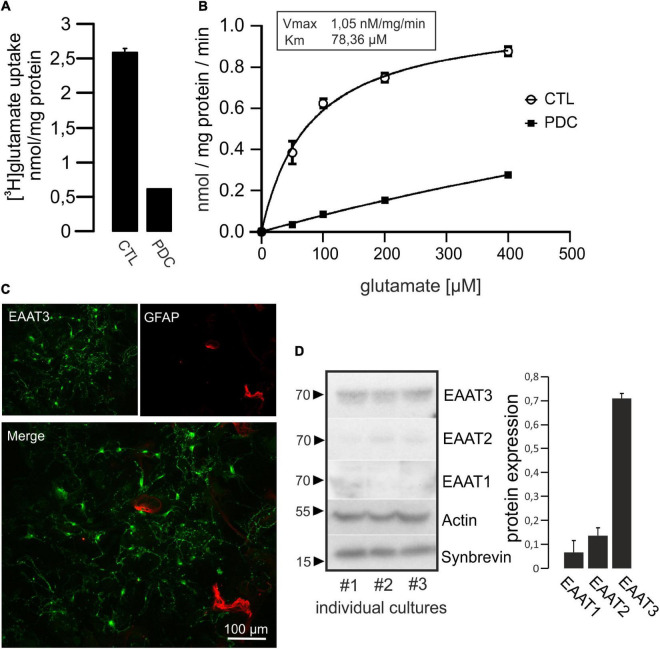
Hippocampal neurons take up [^3^H]glutamate in a specific manner. **(A)** Cultured hippocampal neurons were grown in 24-multiwell dishes for 14 days and incubated for 1 h with radiolabeled glutamate (50 μM). After removal of medium, cells were washed intensively, lysed and radioactivity taken up was calculated by liquid scintillation counting. Values were adjusted to protein content. Specific uptake was evaluated by the addition of 1 mM of the glutamate uptake inhibitor L-*trans*-pyrrolidine-2,4-dicarboxylate (PDC). Uptake of [^3^H]glutamate was strongly inhibited by PDC and therefore regarded as specific. **(B)** Kinetic analysis of glutamate uptake (uptake time 10 min) revealed saturable concentration-dependent transport kinetics (V_max_ and K_m_ values are indicated). Values in **(A,B)** are expressed as means ± SEM from triplicate samples within a single representative experiment repeated two times. **(C)** Neurons were grown on glass cover slips for 14 days, fixed and stained for EAAT3 and GFAP as marker for astrocytes. Staining against EAAT3 and GFAP revealed that the vast majority of cells represented neurons at that culture time. **(D)** Homogenates of cultured hippocampal neurons were subjected to Western blot procedure and probed for the expression of neuronal EAAT3, as well as glial EAAT2 and EAAT1. Actin was used as loading control, Synaptobrevin was used as synaptic neuronal marker. Quantification from three individual cultures revealed that EAAT3 represented the predominant glutamate transporter. Values in **(D)** are expressed as means ± SEM adjusted to Actin from three individual cultures.

Prior to the investigation of putative effects of C3bot preparations on glutamate uptake we tested the capacity of hippocampal neurons to take up glutamate in a specific manner. To this end, cultivated cells were incubated with radiolabeled glutamate. Following incubation with [^3^H]glutamate (total glutamate concentration 50 μM) for 60 min, cells were lysed and taken up glutamate was determined by liquid scintillation counting. Uptake of glutamate by hippocampal neurons was calculated to 2.6 nmol/mg protein (Figure 2A). Incubation with the potent glutamate uptake inhibitor L-*trans*-pyrrolidine-2,4-dicarboxylate (PDC, 1 mM) reduced the uptake by around 75%, confirming specificity of a large proportion of the transport. In a kinetic analysis of uptake we applied glutamate concentrations between 40 and 400 μM (Figure 2B). The maximal transport velocity V_max_ was calculated to 1.05 nmol/mg/min and K_m_ was at 78.36 μM. To visualize the extent of astrocytes present in culture and to estimate the proportion of glial EAAT1 and EAAT2 to the observed uptake we identified astrocytes by GFAP labeling and immunofluorescence imaging. At 14 days *in vitro*, only a minor proportion of GFAP-positive astrocytes was detected compared to the vast majority of EAAT3-positive neurons (Figure 2C). In addition, transporter expression was quantified biochemically. As expected, Western blot analysis revealed a predominant expression of EAAT3, whereas EAAT2 and especially EAAT1 were detected at noticeably lower levels (Figure 2D). Taken together, hippocampal neurons seemed an adequate model to investigate effects of C3 preparations on specific glutamate transport activity in hippocampal cultures predominantly driven by EAAT3.

**FIGURE 3:**
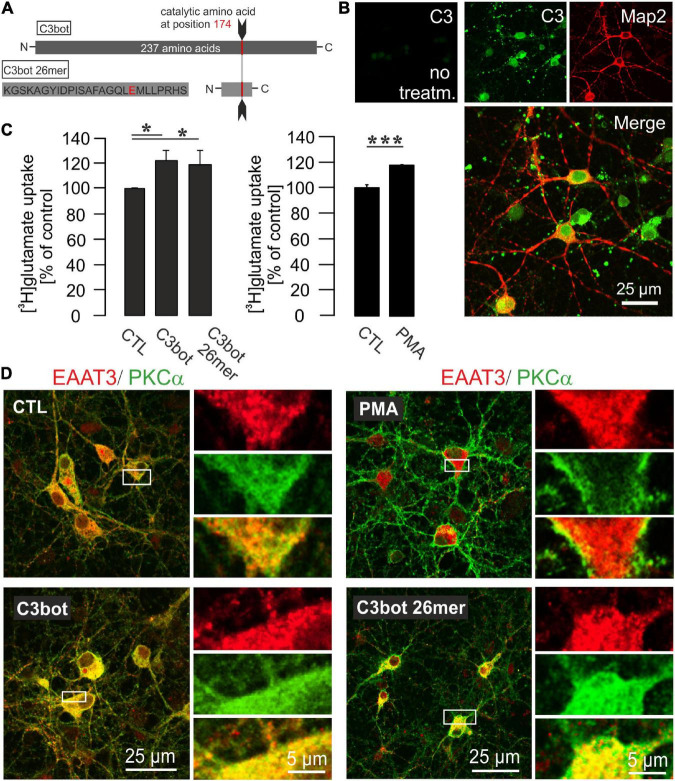
Influence of C3bot, C3bot 26mer, or PKC activation on enhancement of glutamate uptake in hippocampal neurons. **(A)** C3bot 26mer covers the amino acids 156-181 of C3bot full length. Glutamate at position 174 *is* the catalytic amino acid that renders full length C3bot enzymatically active. **(B)** In cultures treated with C3bot full length protein presence of the C3 transferase was visualized after 72 h by a specific antibody directed against C3 protein. The C3bot signal was mainly detected at the somata and Map2-negative processes probably representing axons in a mixture of internalized and surface bound protein. Confocal imaging. **(C)** Hippocampal neurons were incubated with 50 μM [^3^H]glutamate and uptake was measured as described before. Incubation with 300 nM of C3bot or C3bot 26mer for 3 days showed similar effects on uptake regulation. Following incubation with C3bot full length or C3bot 26mer transport activity was increased by C3bot (22%) and, slightly less, by the 26mer (19%). Prior to the uptake procedure, part of the cells were incubated for 30 min with 200 nM of the activator of PKC isoforms PMA. Preincubation with PMA increased the uptake by 19%. In addition, cells were preincubated with dihydrokainate (DHK, 1 mM for 30 min) prior to the uptake procedure to selectively block EAAT2 transport activity. Values therefore represent PDC- and DHK-sensitive counts (analyzed in triplicates) expressed as means ± SEM from 4 to 5 independent experiments. *p ≤ 0.05; ***p ≤ 0.005. **(D)** Confocal imaging of PKCα and EAAT3 distribution in hippocampal neurons after treatment with C3bot, C3bot 26mer, or PMA. In control cells, PKCα showed a marked cytoplasmatic localization, indicative of inactive PKCα. Expression of EAAT3 was also detected throughout the whole cell. Addition of PMA resulted in a shift of PKCα to the plasma membrane characteristic for activation. Conversely, incubation with C3bot or C3bot 26mer did not result in activation of PKCα. Distribution of EAAT3 remained unchanged after either treatment.

**FIGURE 4:**
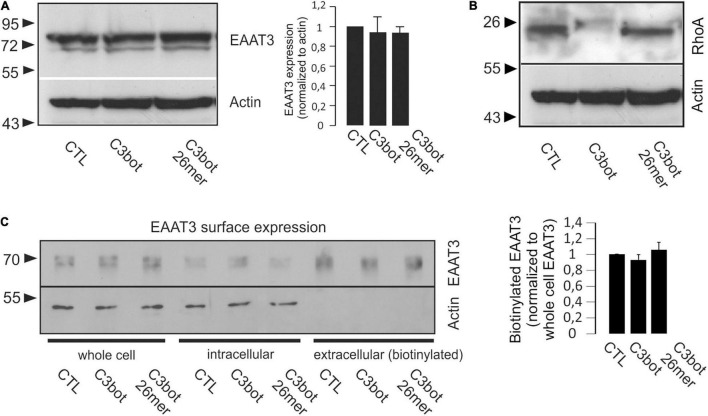
Treatment with C3bot or C3bot 26mer does not alter protein expression and surface location of EAAT3. **(A)** Western blot analysis of EAAT3 expression following treatment of hippocampal neurons for 3 days with 300 nM of C3bot or C3bot 26mer. Quantification revealed that following either treatment, protein levels of EAAT3 remained unchanged after incubation with the C3 preparations. Values are expressed as means ± SEM from three independent experiments. **(B)** ADP-ribosylation of RhoA by C3bot. Homogenates of hippocampal neurons treated as above were subjected to Western blotting analysis and stained for RhoA. Actin served as loading control. Cells treated with C3bot exhibited a complete shift of RhoA to higher molecular weight form indicative of quantitative ADP-ribosylation by enzyme competent C3bot. Also, degradation of inactivate RhoA was observed. **(C)** Surface expression of EAAT3 was analyzed by biotinylation assays. Hippocampal neurons treated with C3bot or C3bot 26mer as above were subjected to biotinylation and subsequent subcellular fractionation into intracellular and extracellular (biotinylated) compartments. Quantification of EAAT3 in the pelleted extracellular fraction revealed no significant changes in surface expression of the transporter. Values are expressed as means ± SEM from three independent experiments.

**FIGURE 5:**
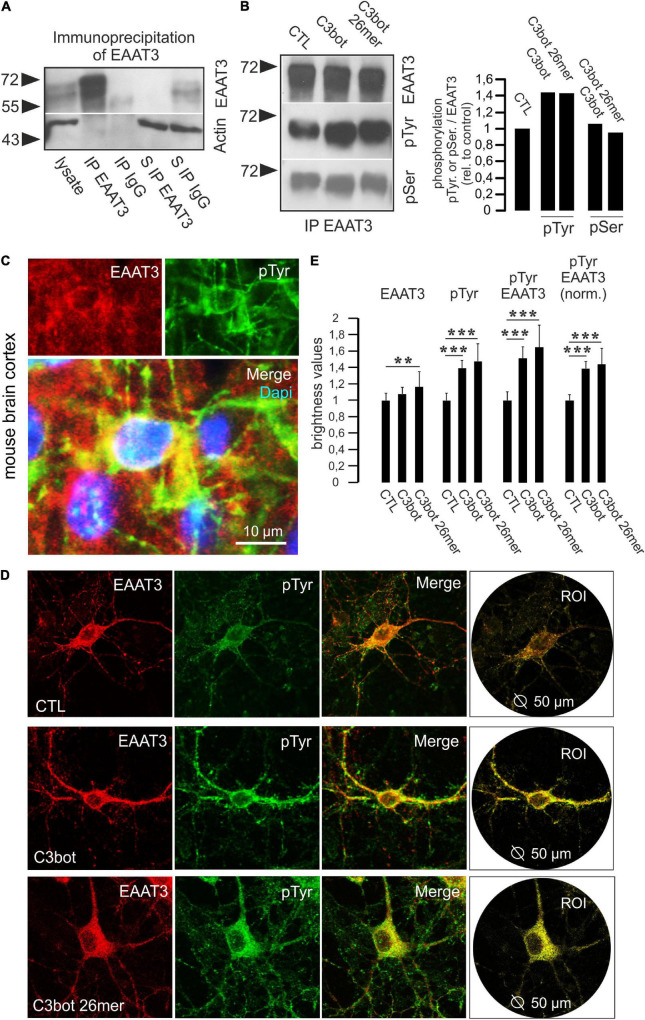
Phosphorylation at tyrosine residues of EAAT3 is mediated by C3bot and 26mer. **(A)** Immunoprecipitation of EAAT3 from hippocampal cell lysates. Hippocampal neurons were lysed and subjected to immunoisolation of EAAT3. Lysates, precipitated fractions and remaining supernatants were probed for EAAT3 expression. Beads coated with rabbit IgG were used for negative control. Actin staining was used for loading control. For detection, light chain-specific secondary antibodies were applied to prevent unwanted detection of heavy chains of the precipitating polyclonal EAAT3 antibody. In the EAAT3 immunoprecipitates (IP) a strong transporter signal was detectable confirming precipitation of transporter by the antibody. In the rabbit IgG fraction no transporter signal was detectable. Clearance of the lysate from the EAAT3 signal following immunoisolation of EAAT3 (supernatant S IP EAAT3) confirmed proper assay function. **(B)** Hippocampal neurons were treated with 300 nM of C3bot or C3bot 26mer for 3 days. Immunoisolates were probed for EAAT3 and phosphorylation of the transporter at tyrosine and serine residues (pTyr, pSer) by phosphor-specific antibodies. Signals were quantified by calculating the ratio of phosphorylation signals to transporter expression and normalized to control conditions. Incubation with C3bot and C3bot 26mer resulted in an increased phosphorylation of tyrosine residues by 40%, phospho-serine signals were largely unchanged. **(C)** Phosphorylation of EAAT3 at tyrosine residues occurs *in vivo*. Cryosections from adult mouse brains were incubated with antibodies against EAAT3 and pTyr. Images depict co-localization of both signals in cortical neurons. **(D)** Phosphorylation of EAAT3 at tyrosine residues. Hippocampal neurons were incubated for 72 h with 300 nM of C3bot or C3bot 26mer. Fixed neurons were stained for EAAT3 and phosphorylated tyrosine. Co-localized signals were quantified by brightness analysis. Red and green fluorescence signals were subtracted and the remaining yellow fluorescence was analyzed within a circular region of interest (ROI) with a diameter of 50 μm centered on the soma of individual neurons. **(E)** Quantification of confocal imaging data. Incubation with both C3bot or C3bot 26mer increased the pTyr signal, peptidic C3bot also had a moderate positive effect on EAAT3 expression. Both treatment regimes resulted in an increased phosphorylation of EAAT3, shown both as total transporter phosphorylation and as values normalized to transporter expression. Values are expressed as means ± SEM from three (CTL and C3bot) or two (C3bot 26mer) independent experiments. CTL: *n* = 110 neurons; C3bot *n* = 102 neurons, C3bot 26mer, *n* = 69 neurons. **p ≤ 0.01; ***p ≤ 0.005.

**FIGURE 6:**
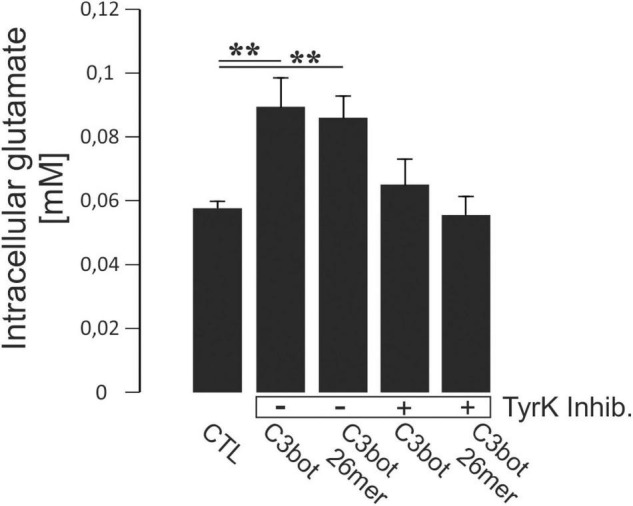
Hippocampal neurons grown for 14 days in culture were incubated with 300 nM of C3bot or C3bot 26mer for additional 3 days with or without the addition of the tyrosine kinase inhibitors Dasatinib (200 nM) and Genistein (20 μM). Cells were then incubated with 50 μM of unlabeled glutamate for 1 h. Prior to the uptake, cells were preincubated with dihydrokainate (DHK, 1 mM for 30 min) to selectively block EAAT2 transport activity. After washing, cells were lysed and intracellular glutamate concentrations were analyzed fluorometrically and adjusted to protein content of each individual well. Neurons incubated with C3bot or C3bot 26mer showed intracellular glutamate concentrations enhanced by 53 and 46%, respectively. These effects were blocked in the presence of tyrosine kinase inhibitors. Values (analyzed in quadruplicates) are PDC- and DHK-sensitive and were calculated from fluorescence units and glutamate standards expressed as means ± SEM from 2 independent experiments. **p ≤ 0.01.

Full-length C3bot has already been shown to increase glutamate uptake in astrocytes (Höltje et al., 2008). These effects turned out to be a specific gene regulatory mechanism and did not result from the strong morphological alterations (stellation) astrocytes undergo in response to inhibition of RhoA by C3bot. C3bot 26mer covers the amino acids 156-181 of the full length sequence (Figure 3A) and has proven to be effective in increasing neuronal process outgrowth *in vitro* as well as to foster functional recovery and regenerative axon re-growth following experimental spinal cord injury in mice (Loske et al., 2012). These previous findings were qualitatively confirmed (Supplementary Figure 1). Hitherto, its effects on neuronal glutamate handling have not been studied. Although full length C3bot neuronal membrane binding and internalization have not completely understood so far intracellularly enriched C3bot can be easily visualized by a specific antibody not available for the peptide (Figure 3B). Transport activity of EAAT3 is known to be highly regulated. Amongst these regulatory factors a main signaling molecule is protein kinase C-alpha (PKCα) which is capable to change transport velocity and plasma membrane localization of EAAT3 (Huang et al., 2006). In this context, it has been demonstrated a convergence between Rho-regulated (and therefore C3bot-sensitive) and PKC signaling pathways (Peng et al., 2011). Moreover, a close association between PKCα and Rho GTPases has been shown (Pang and Bitar, 2005). To investigate C3-mediated alterations in glutamate uptake by hippocampal neurons we incubated cells with 300 nM of C3bot or C3bot 26mer for 72 h and analyzed the amount of glutamate uptake. C3bot increased the specific uptake by around 22% whereas transport activity was enhanced by 19% following incubation with the peptide (Figure 3C, left panel). To take into account that mainly EAAT2 might contribute to some extent to the uptake and therefore to minimize glial transport activity dihydrokainate (DHK) was used in all experimental conditions to specifically block glial EAAT2 activity. In addition, part of the cells under any given condition were also incubated with PDC as shown before to subtract non-specific uptake.

To test for the influence of PKCα on EAAT3-mediated transport activity in our neuronal systems, PKCα was activated by the phorbol ester phorbol 12-myristate 13-acetate (PMA, 200 nM, 20 min). Similar to the effects detected after treatment with C3bot preparations, preincubation of neurons with PMA enhanced glutamate uptake by 19% (Figure 3C, right panel). Confocal imaging confirmed PMA-mediated activation of PKCα by the shift from a cytoplasmatic distribution to a plasma membrane associated form (Figure 3D, right upper panel). Translocation of PKCα to the plasma membrane, on the other hand, could not be evoked by either C3bot or the 26mer, indicating a PKCα-insensitive mode of action (Figure 3D). Under either condition, no obvious redistribution of EAAT3 was detected. So, despite similar effects on glutamate uptake in neurons, effects mediated by C3bot preparations did not seem to base on PKCα activation. In addition to PKCα, activation of other classical PKC isoforms such as PKCγ or novel PKCε as well as unconventional PKCζ was analyzed by immunocytochemistry. However, neither treatment with C3bot nor C3bot 26mer showed an effect on these PKC isoforms as judged by lack of translocation to the plasma membrane (Supplementary Figure 2). Treatment with PMA, on the other hand, had a strong effect on the redistribution of PKCγ and, to a lesser extent, on PKCε, but as expected was ineffective in changing the expression of unconventional PKCζ. Taken together, uptake effects elicited by C3bot and C3bot 26mer do not seem to rely on activation of PKC. To address the cellular mechanisms involved in enhanced glutamate transport, we investigated the expression of EAAT3 after treatment with C3bot full length and peptide. Western blot analysis of whole cell lysates from hippocampal neurons, however, failed to detect changes in overall protein levels of EAAT3 (Figure 4A). To investigate effects of treatment on RhoA expression and ADP-ribosylation, cell lysates were probed for RhoA by Western blotting. Following incubation with C3bot, a complete shift of the RhoA signal to a higher molecular weight form became observable after 72 h of incubation, clearly indicative of C3bot-mediated ADP-ribosylation of RhoA (Figure 4B). Along with this, the RhoA signal was strongly reduced, indicative of the expected proteasomal degradation of RhoA following ADP-ribosylation and inactivation. Due to the lack of enzymatic activity of the 26mer, the RhoA signal remained at the same molecular weight and was largely unchanged. To address putative changes in transporter surface localization that might underlie the enhanced uptake activity, biotinylation experiments were performed. Again, following biotinylation of surface proteins and subsequent subcellular fractionation of hippocampal neurons, no significant alterations in the amount of biotinylated, therefore surface located, EAAT3 were detectable after either treatment regime (Figure 4C).

Since we were unable to detect major changes in total protein expression or transporter localization following incubation with either C3bot or C3 26mer, we aimed to investigate whether direct alterations such as the phosphorylation status of EAAT3 might account for the observed effects on transport activity. Therefore, we performed immunoprecipitation studies to isolate EAAT3 from cell lysates obtained from primary neurons, and incubated the isolated fraction with phospho-specific antibodies. To test for proper assay function, hippocampal cell lysates were incubated with magnetic beads coupled to the polyclonal EAAT3 antibody as well as rabbit IgG for negative control. In the EAAT3-immunoisolates a strong transporter signal could be detected that was absent in the IgG fraction (Figure 5A). Quantitative precipitation of EAAT3 from lysates was confirmed by lack of EAAT3 staining of the remaining supernatant (Figure 5A). We probed EAAT3 immunoprecipitates with phosphotyrosine (pTyr) and phospho-serine (pSer) antibodies to visualize putative changes in transporter phosphorylation. Phosphorylation of tyrosine was upregulated by about 40% in hippocampal neurons, whereas the phospho-serine signal remained largely unchanged (Figure 5B). To quantify the phosphorylation status of EAAT3 on the level of individual neurons we performed co-stainings for EAAT3 and phosphorylated amino acids by immunofluorescence. Due to the fact that both antibodies against EAAT3 and pSer were generated in the same species (rabbit) and the initial IP experiments yielded no effects of treatment on the pSer status of EAAT3 we performed the experiments with respect to phosphorylation at tyrosine residues only. At first, immunohistochemistry on mouse brain sections was performed to show that phosphorylation of EAAT3 at tyrosine residues occurs *in vivo*. Indeed, pTyr signals were found to co-localize with EAAT3 signals in neurons of various brain areas, including the cortex (Figure 5C). To quantify the amount of pTyr co-localizing with the EAAT3 signal following treatment of hippocampal neurons with C3bot and C3bot 26mer, cultures were treated as above and the amount of co-localization was quantified. We chose to perform this analysis at the level of individual neurons to ensure attribution of both signals to cells morphologically identifiable as neurons (Figure 5D). Analysis was restricted to a soma-centered circular region of interest (ROI) with a diameter of 50 μm to exclude putative unspecific effects on protein abundance by the enhanced branching pattern of the more distal neuronal processes mediated by C3bot or C3bot 26mer. Quantification revealed that incubation with C3bot had no effect on EAAT3 transporter expression, whereas C3bot 26mer slightly increased EAAT3 expression by 16% (Figure 5E). Both full length protein and peptide enhanced overall pTyr signals by 40 and 48%, respectively. Co-localization of EAAT3 and pTyr were increased by 50.1% (full length C3bot) and 63% (peptide). More importantly, when normalized to EAAT3 expression the amount of co-localized signals between transporter and pTyr was increased by 39% following C3bot incubation and by 45% following treatment with 26mer.

In order to address directly whether the observed enhanced glutamate uptake following incubation with C3bot full length and peptide resulted from tyrosine phosphorylation we investigated the effect of blocking this process. During incubation with C3bot or C3bot 26mer, neurons were treated with a combination of the established tyrosine kinase inhibitors Dasatinib (200 nM) and Genistein (20 μM). We used a fluorometric assay to determine total intracellular glutamate levels from lysed cells after the uptake assay (glutamate was presented for 1 h at 50 μM in the medium). Incubation with C3bot or C3bot 26mer alone significantly increased intracellular glutamate concentrations by 53 and 46%, respectively (Figure 6). Addition of tyrosine kinase inhibitors completely blocked this effect. These findings add to the previous observation of an increased uptake of (labeled) glutamate into hippocampal neurons following incubation with C3 proteins by also demonstrating higher total intracellular glutamate levels. The sensitivity of this effect to tyrosine phosphorylation further underlines the contribution of this mechanism to an increased neuronal glutamate uptake mediated by C3bot and C3bot 26mer.

In summary, incubation of hippocampal neurons with full length C3bot and C3bot 26mer increased glutamate uptake activity mediated by EAAT3. The effect is very likely duo to an enhanced phosphorylation of EAAT3 at tyrosine residues.

## Discussion

### Phosphorylation of Excitatory Amino Acid Transporter 3 Mediated by C3bot and C3bot 26mer

The current study addresses a novel regulatory mechanism of the neuronal glutamate transporter EAAT3 mediated by C3 proteins. Both full length C3bot and peptidic C3bot^156–181^ (C3bot 26mer) exhibited moderate yet significant effects on glutamate transport in hippocampal neurons by increasing the uptake. In this context, total intracellular glutamate levels were increased. Biochemical and immunocytochemical evidence showed that alterations in the phosphorylation state of EAAT3 might be the underlying mechanism for the increased uptake. Pharmacological interference with tyrosine phosphorylation was able to block the effects of increased uptake. Furthermore, immunofluorescence methods revealed a slight increase in transporter expression following incubation with the C3 peptide. The exact mechanisms of action, however, remain elusive since the classical downstream-pathway of enzymatic inhibition of Rho-dependent signaling cascades can be excluded due to the lack of enzymatic activity. In this context, enzymatic full length protein and enzyme-deficient C3bot peptide must either serve different neuronal pathways that trigger tyrosine phosphorylation at EAAT3 or full length C3bot exhibits a so far unknown enzyme-independent mode of action shared by the peptide. However, a comparable neuronal response in the context of axon outgrowth, synapse formation, regeneration and neuronal plasticity following CNS lesion, as well as functional recovery *in vivo* was already observed after treatment with full length C3bot and C3bot peptides despite the lack enzymatic activity of C3bot peptides (Höltje et al., 2009; Boato et al., 2010; Loske et al., 2012). On the other hand, trophic effects on glial morphology and also glutamate handling by astrocytes seem to strictly rely on the enzymatic inhibition of Rho proteins and cannot be elicited by enzyme deficient C3 preparations (Höltje et al., 2005, 2008). The observed alterations in total intracellular glutamate levels mediated by C3bot and the peptide appeared to be larger than the pure uptake effects. Therefore, it can not be excluded that C3 proteins exert additional effects on neuronal glutamate turnover like reverse transport or degradation mechanisms. Part of the differences, however, between the effects on the uptake of labeled glutamate and the more pronounced increase in total glutamate might also arise from the different experimental paradigms using either radiolabeled glutamate or fluorometric (enzymatic) detection of unlabeled glutamate.

What regulatory mechanisms mediated by C3 proteins might be involved in the increased phosphorylation of EAAT3? A recent study showed that activation of the C3bot target RhoA by amphetamine in cultured noradrenergic neurons that also carry EAAT3 resulted in a decreased glutamate uptake that was, however, due to an enhanced endocytosis of EAAT3 (Underhill et al., 2020). Since C3bot and C3bot 26mer obviously do not interact with regulatory modulations controlling trafficking of EAAT3 to the neuronal surface as described for constitutive Rab11- or adapter protein-2-dependent endocytosis and recycling (González et al., 2007; Saha et al., 2021) or stimulated trafficking by PKCα (this paper, González et al., 2003), a direct functional regulation seems to occur. In earlier studies it was shown that PKCα-dependent phosphorylation of serine 465 enhanced isoflurane-induced activity of EAAT3, but again required EAAT3 redistribution to the plasma membrane (Huang et al., 2006, 2011). In our experiments incubation with C3bot preparations did neither result in PKCα activation and subsequent EAAT3 redistribution nor seemed serine phosphorylation to be involved. As observed, incubation with C3 proteins (followed by inactivation of Rho proteins at least in the case of enzyme-competent full length protein) results in an increased phosphorylation of EAAT3, therefore inactivation of phosphatases or activation of kinases represent putative signaling pathways. Blockage of Rho-signaling by C3 proteins or downstream effectors like ROCK or mDia would result in decreased kinase activity and therefore represent an unlikely mechanism. Besides the well-characterized Rho protein downstream effector kinases that are mainly involved in phosphorylation of cytoskeletal proteins knowledge about interaction of Rho proteins with phosphatases is sparse. An interaction of activated RhoA with the inositol 5-phosphatase SHIP2 has been described that is involved in cell polarity and migration (Kato et al., 2012). To our knowledge, no phosphorylation pathway has been described so far that regulates EAAT3 activity without affecting its localization. Future work has to elucidate these pathways mediated by full length as well as peptidic C3bot. In case of C3bot 26mer, neuronal EAAT3 expression was slightly enhanced as observed by immunofluorescence. This was not reflected by the biochemical experiments carried out on whole cells and biotinylation experiments. We can only speculate that this discrepancy resulted from the different methods used (immunofluorescence on individual neurons versus Western blot of whole cultures). In line with this, undetectable changes in total protein by Western blotting might reflect the fact that EAAT3 expression in all cell types, including glial cells that might express smaller transporter amounts, was looked at.

### Functional Implications

Generally, an increased glutamate clearance (stimulated by C3 preparations) from the extracellular space might be favorable under certain pathophysiological circumstances, e.g., spinal cord injury (SCI) in order to prevent further neuronal damage by excessive extracellular glutamate concentrations (Park et al., 2004). Given the fact that glutamate clearance mediated by EAAT3 serves various functions under both physiological and pathophysiological circumstances (Bianchi et al., 2014) interference with its function might, however, result in different outcomes. Generally, surface EAAT3 localizes rather perisynaptically than in the synaptic cleft of excitatory synapses and one of its function is considered to limit NMDAR activation by glutamate spillover between synapses (Diamond, 2002). Further evidence for the physiological function of EAAT3 in controlling synaptic strength and plasticity by modulating long-term potentiation (LTP) in the hippocampus stems from CA1 pyramidal cells lacking EAAT3 transport activity. Experiments showed that EAAT3 buffers glutamate release during synaptic events and reduces the recruitment of NMDAR subunits thereby facilitating LTP (Scimemi et al., 2009). Also, GABAergic neurotransmission and neuronal glutathione synthesis depend on EAAT3 activity (Sepkuty et al., 2002; Aoyama et al., 2006).

More recently, accumulating evidence has shown that altered EAAT3 function is associated with several neurological or psychiatry spectrum disorders. Many of these studies focus on genetic findings that document interference with transporter expression or localization. Amongst them are the obsessive-compulsive disorder state (Zike et al., 2017; Delgado-Acevedo et al., 2019; Underhill et al., 2019) and schizophrenia (Afshari et al., 2015). Hitherto, despite the accumulating evidence for the contribution of EAAT3 to the described physiological mechanisms and pathophysiological conditions the lack of a specific pharmacological inhibitor of EAAT3 has prevented a deeper understanding of its exact role in these processes. However, the advent of newly discovered imidazo[1,2-*a*]pyridine-3-amine compounds to selectively inhibit EAAT3 activity might improve future research (Wu et al., 2019). It is tempting to speculate that the observed neuroprotective effect (early onset of functional recovery and reduced lesion size) of treatment with C3bot 29mer and 26mer peptides following spinal cord injury in mice (Boato et al., 2010; Loske et al., 2012) might at least partially result from an increased glutamate uptake by neuronal EAAT3 to prevent excessive glutamate excitotoxicity often observed after injury. This might, however, include the glial transport systems following treatment with enzyme-competent full length C3bot (mainly GLT-1/EAAT2) that have been shown to be upregulated *in vitro* by C3bot (Höltje et al., 2008).

Taken together, we demonstrate a novel C3bot/C3bot 26mer-dependent tyrosine phosphorylation of the neuronal glutamate transporter EAAT3 by a so far unknown pathway that is very likely to result in an enhanced uptake activity.

## Data Availability Statement

The original contributions presented in this study are included in the article/Supplementary Material, further inquiries can be directed to the corresponding author/s.

## Ethics Statement

Animal housing as well as all experiments using animal-derived tissue/cells were performed in accordance with institutional (Charité–Universitätsmedizin Berlin, Germany), local (LaGeSo, Berlin) and national guidelines (German Animal Welfare Act).

## Author Contributions

JP and MH performed the experiments and designed the study. AR, IJ, SB, and GA-H contributed to the conception and design of the study. MH wrote the first draft of the manuscript. All authors contributed to manuscript revision, read, and approved the submitted version.

## Conflict of Interest

The authors declare that the research was conducted in the absence of any commercial or financial relationships that could be construed as a potential conflict of interest.

## Publisher’s Note

All claims expressed in this article are solely those of the authors and do not necessarily represent those of their affiliated organizations, or those of the publisher, the editors and the reviewers. Any product that may be evaluated in this article, or claim that may be made by its manufacturer, is not guaranteed or endorsed by the publisher.

## Supplementary Material

Supplementary Figure 1 Increased neuronal outgrowth of hippocampal neurons following incubation with C3bot or C3 26mer. Hippocampal neurons were treated for 3 days with 300 nM of full length C3bot or peptidic C3bot 26mer, fixed and stained for Map2 as dendritic marker protein. Both C3bot and C3bot 26mer enhanced neuronal process outgrowth as visualized by immunofluorescence.

Supplementary Figure 2 Hippocampal neurons grown for 14 days in culture were incubated with either 300 nM of C3bot or C3bot 26mer (3 days) or 200 nM of PMA for 20 min. Cells were fixed and stained against classic protein kinase C gamma (PKCγ), novel protein kinase C epsilon (PKCε), and unconventional protein kinase C zeta (PKCζ). Readout of activation of PKCs was a shift to the plasma membrane. Under control conditions, neurons showed a mainly cytoplasmatic PKC expression, being most prominent for PKCγ, followed by PKCζ and only very weak for PKCε. Incubation with C3bot full length or peptide had no effects on PKC localization, irrespective of the isoform. PMA, on the other hand, strongly activated PKCγ and, to some extent, PKCε in some cell somata. As expected for unconventional PKC isoforms, PKCζ was unaffected by PMA.
