# Supplementary figures and images for: Enhancement of Phosphorylation and Transport Activity of the Neuronal Glutamate Transporter Excitatory Amino Acid Transporter 3 by C3bot and a 26mer C3bot Peptide
Source: Front Cell Neurosci. 2022 Jun 15;16:860823. doi: 10.3389/fncel.2022.860823 (PMC9240211; doi:10.3389/fncel.2022.860823)

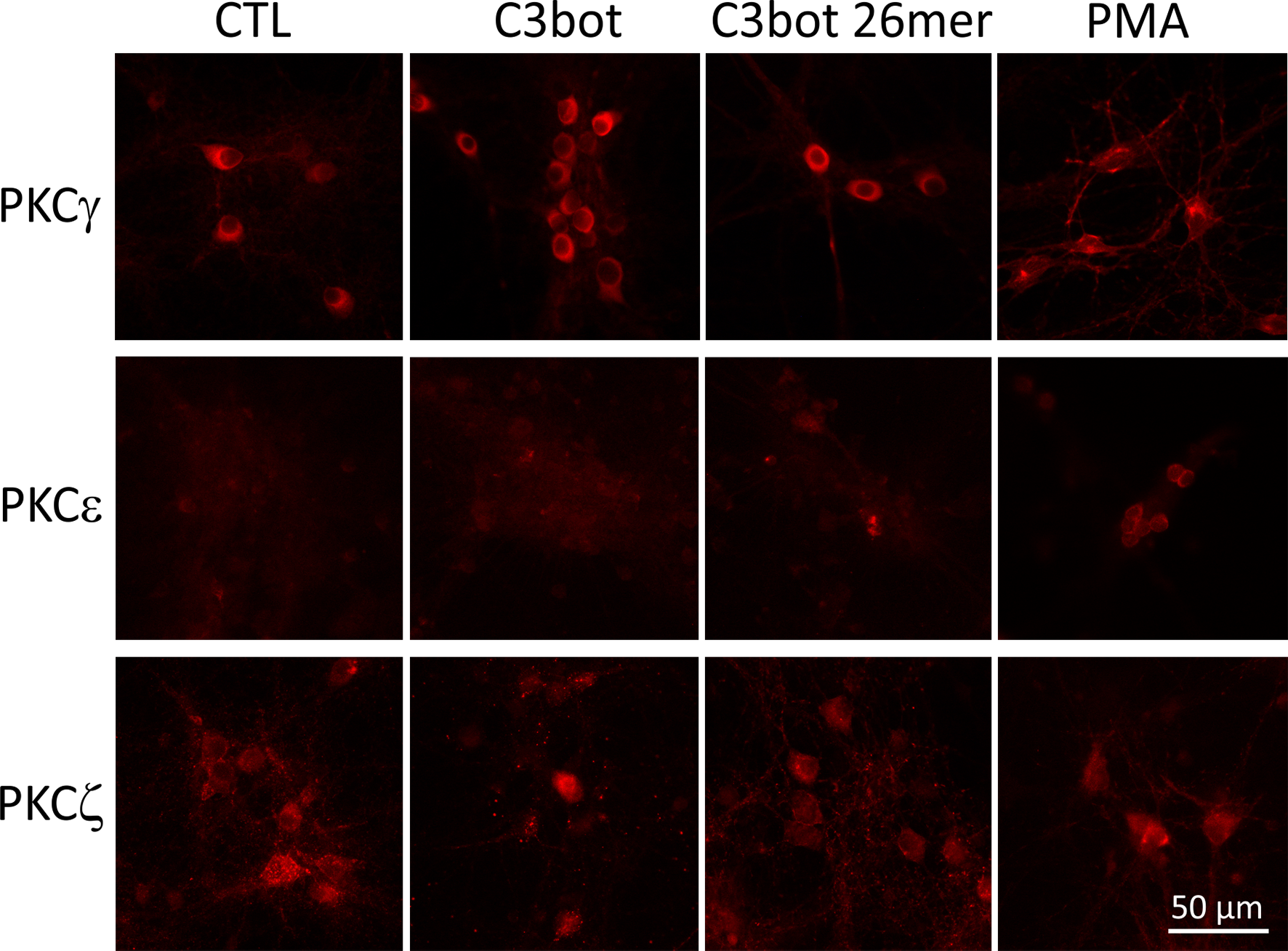

Supplement: Supplementary Figure 2 — Hippocampal neurons grown for 14 days in culture were incubated with either 300 nM of C3bot or C3bot 26mer (3 days) or 200 nM of PMA for 20 min. Cells were fixed and stained against classic protein kinase C gamma (PKCγ), novel protein kinase C epsilon (PKCε), and unconventional protein kinase C zeta (PKCζ). Readout of activation of PKCs was a shift to the plasma membrane. Under control conditions, neurons showed a mainly cytoplasmatic PKC expression, being most prominent for PKCγ, followed by PKCζ and only very weak for PKCε. Incubation with C3bot full length or peptide had no effects on PKC localization, irrespective of the isoform. PMA, on the other hand, strongly activated PKCγ and, to some extent, PKCε in some cell somata. As expected for unconventional PKC isoforms, PKCζ was unaffected by PMA. [file Image_2.TIF]
